# Prognostic analysis of tumour angiogenesis, determined by microvessel density and expression of vascular endothelial growth factor, in high-risk primary breast cancer patients treated with high-dose chemotherapy

**DOI:** 10.1038/sj.bjc.6603875

**Published:** 2007-07-03

**Authors:** Y Nieto, J Woods, F Nawaz, A Baron, R B Jones, E J Shpall, S Nawaz

**Affiliations:** 1 Department of Stem Cell Transplantation and Cellular Therapy, University of Texas MD Anderson Cancer Center, Houston, TX, USA; 2 Department of Pathology, University of Colorado, Denver, CO, USA; 3 Department of Biostatistics, University of Colorado, Denver, CO, USA

**Keywords:** high-risk breast cancer, angiogenesis, microvessel density, vascular endothelial growth factor, prognostic factor, high-dose chemotherapy

## Abstract

In contrast to early breast cancer, the prognostic effect of tumour angiogenesis in tumours with advanced axillary spread has been less studied. We retrospectively analysed the effect of microvessel density (MVD) and vascular endothelial growth factor (VEGF) by immunohistochemistry on the outcome of 215 patients treated uniformly within prospective trials of high-dose chemotherapy for 4–9 and ⩾10 positive nodes, and followed for a median of 9 (range 3–13) years. Microvessel density was associated with epidermal growth factor receptor (EGFR) expression (*P*<0.001) and tumour size (*P*=0.001). Vascular endothelial growth factor overexpression (51% of patients) was associated with overexpression of EGFR (*P*=0.01) and HER2 (*P*<0.05), but not with MVD (*P*=0.3). High MVD was associated with worse relapse-free survival (74 *vs* 44%, *P*<0.001) and overall survival (76 *vs* 44%, *P*<0.001). Vascular endothelial growth factor overexpression had no effect on outcome. Multivariate analyses showed a prognostic effect of MVD independently of other known prognostic factors in this patient population. In conclusion, tumour angiogenesis, expressed as MVD, is a major independent prognostic factor in breast cancer patients with extensive axillary involvement.

High-risk primary breast cancer (HRPBC) is characterised by extensive axillary involvement, usually defined as four or greater positive nodes. Although still curable, this stage of the disease presents a 50% or greater risk of metastatic relapse despite modern multimodal management. Identification of relevant new biologic targets in this population may allow for improvement of therapeutic outcome.

One such type of target might be those involved in tumour angiogenesis. Angiogenesis promotes tumour growth and metastasis through perfusion of the tumour, facilitation of transport of tumour cells in the bloodstream, and reciprocal paracrine stimulation between endothelial and tumour cells (Folkman, 1990; Fidler and Ellis, 1994). Angiogenesis is part of a complex scenario that also includes tumour suppressor gene and oncogene pathways, adhesion molecules, apoptosis, and the immune system. The angiogenic status of a tumour depends on the balance between proangiogenic and antiangiogenic factors that determines a state of tumour dormancy, which can last for a long time in breast cancer, or a predominance of angiogenesis, which appears to trigger disease progression or relapse.

The vascular endothelial growth factor (VEGF) is one of the most potent inducers of angiogenesis (Ferrara *et al*, 2003). Although it is produced by many different cells, its mitogenic activity mainly focuses on endothelial cells. Vascular endothelial growth factor and its tyrosine kinase receptors are subjected to regulation by tissue oxygen conditions as well as by oncogenes, such as HER2, or tumour-suppressor genes.

Many studies, but not all, have suggested a prognostic effect of angiogenesis in early breast cancer, defined by no or limited axillary involvement. A meta-analysis detected an overall prognostic adverse effect of microvessel density (MVD) among node-negative patients (Uzzan *et al*, 2004). The discrepancies among the studies may be the result of methodological differences or heterogeneity of patients and treatments. In consequence, it has been recommended that angiogenesis markers are not to be used as basis for making clinical decisions (Hayes *et al*, 2001). The College of American Pathologists has stated that further study of quantification of tumour angiogenesis is still required to demonstrate its prognostic value in breast cancer (Fitzgibbons *et al*, 2000).

Furthermore, less is known about the prognostic effect of angiogenesis in patients with advanced axillary involvement, who present a higher likelihood of micrometastatic spread at the time of diagnosis, and, thus, may constitute a different biological scenario from node-negative tumours. We studied the effect of tumour angiogenesis in HRPBC by immunohistochemical analysis of MVD and VEGF in samples collected retrospectively from patients who were treated uniformly within high-dose chemotherapy (HDC) trials and subjected to long-term follow-up.

## MATERIALS AND METHODS

### Patient population

This study adhered to the NCI-EORTC guidelines for tumour marker prognostic studies (McShane *et al*, 2005). We retrospectively analysed tumour samples from patients who were prospectively enrolled in phase II and III trials of HDC for HRPBC at the University of Colorado between 1990 and 2001. A total of 234 patients were accrued in these studies, which were open to patients with 4–9 positive axillary nodes (*n*=102) (Bearman *et al*, 1997; Moore *et al*, 2007) and 10 or greater positive nodes (*n*=132) (Nieto *et al*, 2004; Peters *et al*, 2005). Those trials, as well as the current retrospective review of tumour blocks, were approved by the Cancer Center Protocol Review Committee and the Institutional Review Board. All patients gave written informed consent before study entry. Eleven patients (4.7% of the total enrolment) who died from HDC-related complications were excluded, leaving 223 patients eligible for this analysis. Tumour samples could be obtained from 215 of those 223 patients.

Inclusion and exclusion criteria of those trials were similar, except for the required number of axillary nodes involved. The studies required adequate visceral organ function. Pretransplantation staging tests included computed tomographic scans of the head, chest, abdomen, and pelvis, bone scans, and bone marrow biopsies. Patients received HDC within 6 months of their primary surgery (mastectomy or lumpectomy with negative margins). Patients received four cycles of doxorubicin-containing chemotherapy before HDC. Absence of relapse during pretransplantation chemotherapy was required. Following collection of haematopoietic progenitor cells, patients received high-dose cyclophosphamide/cisplatin/carmustine, as described (Peters *et al*, 2005). Subsequently, unselected haematopoietic progenitor cells were infused, and granulocyte colony-stimulating factor was administered until neutrophil recovery. Post-transplantation treatment included locoregional radiotherapy upon platelet recovery. Tamoxifen was prescribed for 5 years to patients with hormone receptor-positive tumours.

### Immunohistochemical analyses

Paraffin-embedded blocks containing whole-tumour sections from the surgical specimens were retrospectively obtained from the referring institutions. All tumour samples were acquired before initiation of any chemotherapy. Intratumoural microvessels were identified by immunostaining of formalin-fixed paraffin-embedded sections with pan-endothelial anti-CD31 monoclonal antihuman mouse antibody (clone JC/70A; Dako, Carpinteria, CA, USA) in a 1 : 40 dilution and overnight incubation. The invasive tumour component was identified on haematoxylin- and eosin-stained sections. Intratumoural MVD was then determined as described previously (Weidner *et al*, 1991). The area of most intense neovascularisation was selected by scanning on low magnification (× 10–100), preferentially on the peripheral tumour margins. Only the vascularity of tumour areas considered viable, that is, non-necrotic, was taken into account. Subsequently, individual microvessels were counted on a × 400 field. Any brown-staining endothelial cell, containing a visible nucleus, and clearly separate from adjacent microvessels, tumour cells and other connective-tissue elements, was considered a single, countable microvessel, without requirement for a lumen or the presence of erythrocytes. The microvessels were counted in a 0.74-mm^2^ area (i.e., a × 400 field). Each patient's microvessel count was the average of two separate counts by two different pathologists (SN and JW) who remained blind to patient outcome.

Vascular endothelial growth factor immunostaining was performed on diagnostic sections using mouse monoclonal antibody anti-VEGF (C-1; Santa Cruz Biotechnology, Santa Cruz, CA, USA). Vascular endothelial growth factor immunoreactivity was scored as 0, 1+, 2+, or 3+, according to staining intensity (Toi *et al*, 1994). A tumour area with any degree of staining was scored as VEGF-positive. Tumours with VEGF-positive and VEGF-negative areas were assigned the score of the area with strongest staining.

Immunohistochemical analyses of HER2 and p53 used the monoclonal antibodies CB11 (Vantana Medical Systems, Tucson, AZ, USA) and DO7 (BioGenex, San Ramon, CA, USA), respectively. The immunohistochemical analysis of epidermal growth factor receptor (EGFR) employed the murine monoclonal antibody 31G7 (Zymed Laboratories, San Francisco, CA, USA) (1 : 100 dilution). Staining intensity was evaluated as 1+ (weak), 2+ (moderate), or 3+ (strong). Cases were graded based on the overall proportion of cells stained with moderate or strong intensity: 0, 0% cells; 1+, 1–33%; 2+, 34–66%; 3+, 67–100%. Cases with ⩾1+ membranous staining for EGFR were considered positive. Cases with any degree of p53 nuclear staining were considered positive. We considered HER2-positive those cases with 2+ (weak complete membrane staining in >10% of cancer cells) or 3+ grading (intense complete membrane staining in >10% of cancer cells). All immunostained slides were reviewed by the same pathologist (SN), who was blinded to patient outcome.

### Statistical methods

Associations between categorical and continuous variables were assessed using the *χ*^2^ or Fisher's exact test and Student's *t*-test, respectively. Median follow-up times were estimated among surviving patients. Relapse-free survival (RFS) time was defined as the time from the administration of HDC to documented relapse (local, contralateral, or distant) or to death without relapse. Overall survival (OS) time was defined as the time from HDC to death from any cause. All survival times were analysed using the Kaplan–Meier method (Kaplan and Meier, 1958). The log-rank test was used to study the association of the potential prognostic variables with survival times (Peto and Peto, 1971). The 25th, 50th, and 75th percentile of the microvessel counts were evaluated as potential prognostic cutoffs.

Multivariate analyses of RFS and OS used proportional hazards Cox regression models (Cox, 1972). These models included the variables previously identified as independent prognostic factors in this population: nodal ratio (i.e., no. of involved axillary nodes/no. of dissected nodes), combined oestrogen (ER) and progesterone receptor (PR) status, the pathologic tumour size and HER2 (Nieto *et al*, 1999, 2000). The remaining variables studied, such as age, menopausal status, family history of breast cancer, histologic grade, tumour ploidy, S-phase fraction, multifocality, vascular invasion, lymphatic invasion, extensive intraductal component, or p53 status, which lacked an independent prognostic effect, were excluded from the multivariate analyses. Likewise, of all the axillary node-related variables previously analysed (nodal ratio, absolute number of involved nodes, presence of involved nodes greater than 2 cm, and extracapsular extension), only the nodal ratio was independently associated with outcome, and included in the multivariate models.

The probability of relapse after HDC for HRPBC, based on a linear regression model with the independent variables, can be expressed by the following equation (Nieto *et al*, 1999):

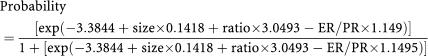


A simpler scoring system was subsequently derived from the previous formula:

Score=(nodal ratio × 3.05)+(tumour size × 0.15)–(ER/PR × 1.19).

In both formulae, tumour size is entered in centimetres, and ER/PR status is assigned ‘1’ if positive (i.e., if ER and/or PR are positive) and ‘0’ if negative (i.e., if both ER and PR are negative). The patient is assigned the high or low risk of relapse category if the score is ⩾2.41 or <2.41, respectively, with highly significant differences in RFS (*P*<10^−5^) and OS (*P*<10^−5^) between both groups. The optimal cutoff score of 2.41, as identified in the receiver operating characteristic curves, conferred on the model a sensitivity of 0.6, a specificity of 0.88, a positive predictive value of 0.65, a negative predictive value of 0.86, and an accuracy of 83%. The prognostic value of this score was confirmed externally (Nieto *et al*, 1999) and prospectively (Nieto *et al*, 2004).

The significance of the current multivariate models was evaluated with the likelihood ratio test. Individual coefficients were tested using Wald's statistic. The proportionality assumption for the variables was assessed with Kaplan–Meier curves. All *P*-values presented are two-tailed. All statistical analyses were carried out using Statview 5.01 software (SAS Institute, Cary, NC, USA).

## RESULTS

**Figure 1:**
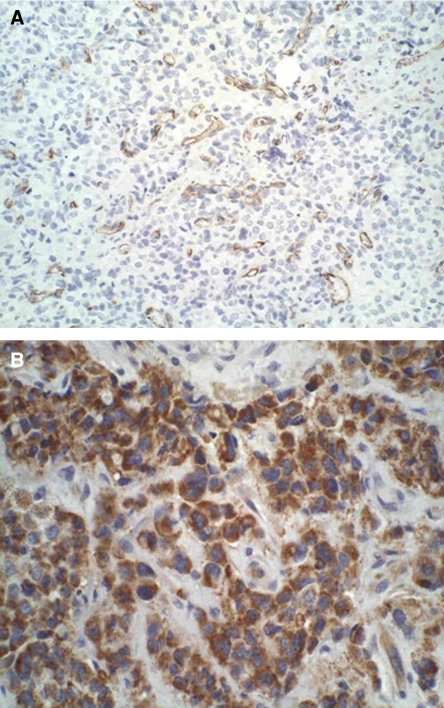
Immunohistochemical staining. (**A**) Intratumour CD31-stained microvessels. (**B**) VEGF staining.

We studied specimens from 215 patients (96% of the eligible population of 223 patients) (Table 1). CD31 staining of tumour vessels is shown in Figure 1A. The median microvessel count was 8 per field (range, 2–27), with low (6%) interobserver variation. The microvessel count was higher in tumours overexpressing EGFR than in EGFR-negative tumours (11.5 *vs* 6; *P*<0.001), and in tumours >2 cm than in those ⩽2 cm (10 *vs* 6.5; *P*=0.001). In contrast, we did not observe an association between MVD and HER2 status (median microvessel counts for HER2-positive and HER2-negative tumours of 8 and 7, respectively, *P*=0.5), nodal status (expressed as either the nodal ratio (*P*=0.8), or the absolute number of positive nodes (*P*=0.4)), p53 status (*P*=0.9), or histologic grade (*P*=0.8). Finally, there was a nonsignificant trend for a higher microvessel count in ER/PR-negative than in ER/PR-positive tumours (8.5 *vs* 7; *P*=0.09).

Intratumoural VEGF expression was detected in 51% of the patients (Figure 1B). Its staining intensity was graded as 1+ in 29 (26%) patients, as 2+ in 44 (40%) patients, and as 3+ in 37 (34%) patients. No correlation was seen between MVD and VEGF expression (*P*=0.3). Vascular endothelial growth factor overexpression was detected in 56% of the patients with HER2-positive tumours, compared with 39% of those with HER2-negative tumours (*P*=0.04). Likewise, 61% of patients with EGFR-positive tumours coexpressed VEGF, compared with 39% of those with EFGR-negative tumours (*P*=0.01). Vascular endothelial growth factor was not associated with the nodal ratio (*P*=0.8), the absolute number of positive nodes (*P*=0.6), tumour size (*P*=0.6), ER/PR status (*P*=0.6), p53 status (*P*=0.9), or histologic grade (*P*=0.7).

### Prognostic analyses

**Figure 2:**
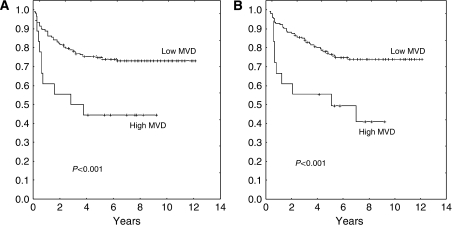
Outcome according to MVD, according to a cutoff 14 microvessels (75th percentile). (**A**) Relapse-free survival; (**B**) overall survival.

We identified a prognostic cutoff count of 14 microvessels (75th percentile). The RFS rates in patients with low and high MVD were 74% (95% confidence interval (CI), 67–80%) and 44% (95% CI, 36–51%), respectively (*P*<0.001) (Figure 2A). Their respective OS rates were 76% (95% CI, 69–82%) and 44% (95% CI, 36–51%) (*P*<0.001) (Figure 2B).

**Figure 3:**
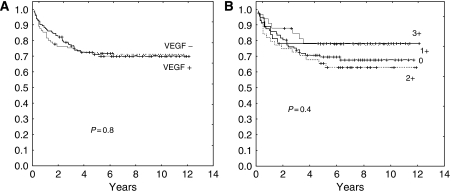
Lack of effect of VEGF expression on RFS. (**A**) Expressors *vs* non-expressors (*P*=0.8). (**B**) According to semiquantitated grading (*P*=0.4).

In contrast, the groups with VEGF-positive and VEGF-negative tumours did not have significantly different RFS (71.5 *vs* 70%, *P*=0.8, Figure 3A) or OS (75 *vs* 70%, *P*=0.4). Analysing VEGF expression according to its semiquantitated grading found no significant outcome differences between the groups with 0, 1+, 2+, or 3+ staining, with RFS rates of 69, 79, 64, and 78%, respectively (*P*=0.4) (Figure 3B).

### Multivariate analyses

Multivariate models included MVD, HER2, and the three independent clinical variables, nodal ratio, tumour size, and ER/PR status. Model 1 included the clinical variables combined as the score. Model 2 analysed all variables separately. Both models showed the independent prognostic effect of MVD on RFS and OS (Table 2).

**Figure 4:**
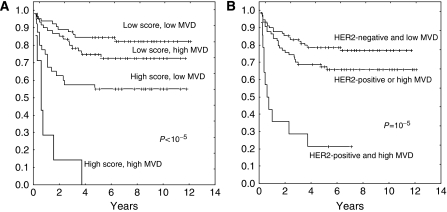
Relapse-free survival combining MVD and other independent prognostic variables. (**A**) Analysis of MVD (cutoff, 14 microvessels – 75th percentile) and the predictive score (cutoff score, 2.41). (**B**) Analysis of MVD and HER2 status.

The combined analysis of MVD and the clinical score offered a highly significant separation of four prognostic categories (*P*<10^−5^) (Figure 4A). Patients with a high score and a low MVD had significantly better RFS than those with a high score and a high MVD (55 *vs* 0%, *P*=0.005). Within the low-score subgroup, however, the differences between patients with low and high MVD approached but did not reach the level of statistical significance (83 *vs* 74%, *P*=0.09).

Combining MVD and HER2 resulted in the following four prognostic groups (*P*<0.0001): (1) HER2 negativity and low MVD (78% RFS), (2) HER2 negativity and high MVD (67% RFS), (3) HER2 positivity and low MVD (66% RFS), and (4) HER2 positivity and high MVD (21% RFS). Merging groups 2 and 3 results in a three-group classification based on the presence of 0, 1, or 2 of those poor-risk features (*P*=0.00001) (Figure 4B).

## DISCUSSION

In this study, we found that a highly angiogenic phenotype, determined by MVD, was a powerful adverse prognostic factor in our group of 215 patients with HRPBC treated with uniform multimodal treatment including HDC. The effect of MVD was independent of other known prognostic factors in this patient population. Tumour microvessels were counted separately by two different pathologists following the international consensus guidelines on angiogenesis quantification (Vermeulen *et al*, 1996), with low interobserver variability.

Although HER2 signaling is believed to promote tumour angiogenesis (Kumar and Yarmand-Bagheri, 2001), we found no association between HER2 overexpression and MVD, in keeping with previous results (Vogl *et al*, 2006). In contrast, others observed an association of MVD and HER2 overexpression in small series of patients with node-negative disease (Koukourakis *et al*, 2003). We noted a significant association between HER2 and VEGF, as did previous studies of mixed populations with node-negative and node-positive tumours (Konecny *et al*, 2004; Linderholm *et al*, 2004). We considered it important that the prognostic effects of tumour angiogenesis and HER2 in our study were mutually independent, suggesting the potential benefit of their concurrent targeting in this population. The feasibility of dual VEGF and HER2 targeting with bevacizumab and trastuzumab has been recently shown (Pegram *et al*, 2004), and could be tested in HRPBC patients.

Another interesting observation was the association between MVD or VEGF and EGFR. Activation of EGFR signaling upregulates VEGF and activates angiogenesis in human brain cancer cells (Goldman *et al*, 1993). An association of EGFR with MVD in breast cancer was previously suggested in a small study of 45 patients (De Jong *et al*, 1998).

In contrast to MVD, we did not observe any significant prognostic effect of VEGF, one of the most potent inducers of angiogenesis. As such, it constitutes a major target for strategies aiming at disrupting angiogenesis. Indeed, the most successful antiangiogenic intervention to date has been the use of bevacizumab in metastatic breast cancer (Miller *et al*, 2005), colorectal cancer (Hurwitz *et al*, 2004), and non-small-cell lung cancer (Sandler *et al*, 2006). However, its usefulness as a potentially informative prognostic marker is a separate issue from its value as a therapeutic target. Similar to previous observations in breast cancer (Lantzsch *et al*, 2002; Chhieng *et al*, 2003), we observed no significant association between VEGF expression and MVD, supporting the notion that multiple angiogenic factors, besides VEGF, play a role in the angiogenic process. Redundancy is a major characteristic of tumours, and different angiogenesis markers may be relevant in different phases of development.

Our study has several potential limitations. One is the inherent bias in all retrospective studies of archival tissue which may be lessened in this study by the high sample collection rate. Another limitation is the fact that all patients analysed received HDC, which is not currently part of the standard management of HRPBC. Although significant superiority of HDC over standard-dose chemotherapy has been seen in some randomised trials (Roché *et al*, 2001; Nitz *et al*, 2005), several other studies have not shown any appreciable outcome differences (Tallman *et al*, 2003; Leonard *et al*, 2004; Coombes *et al*, 2005; Peters *et al*, 2005; Moore *et al*, 2007). In addition, our analysis excluded those patients who relapsed during pretransplantation chemotherapy and never received HDC, which could represent a selection bias. However, this very poor prognosis subgroup represented only 2% of all patients enrolled in those trials (Peters *et al*, 2005).

In conclusion, tumour angiogenesis, expressed as MVD but not as VEGF, was found to be a major independent prognostic factor in patients with HRPBC treated with HDC. These results support the evaluation of antiangiogenic interventions in this high-risk population.

## Figures and Tables

**Table 1:** Patient demographics (*n*=215)

**Characteristic**	**No. of patients (%)**
Age (years): median, range	49, 28–67
	
*Menopausal status*	
Pre/perimenopausal	123 (57)
Postmenopausal	92 (43)
	
*Tumour size (cm)*	
⩽2	59 (28)
>2–5	91 (42)
>5	65 (31)
	
*ER/PR status* a	
Negative	90 (42)
Positive	125 (58)
	
*No. of involved nodes*	
4–9	90 (42)
10–20	83 (39)
>20	42 (19)
	
No. of dissected nodes: median, range	19 (10–46)
Nodal ratio (no. of involved nodes/no. of dissected nodes): median, range	0.55, 0–1
	
*Predictive score* b	
Low (<2.41)	151 (70)
High (⩾2.41)	64 (30)
	
*Histologic grade*	
1–2	78 (36)
3	137 (54)
	
*HER2 status*	
Negative	124 (58)
Positive	91 (42)
	
*p53 status*	
Negative	96 (66)
Positive	50 (34)
Undetermined	69
	
*EGFR status*	
Negative	124 (58)
Positive	91 (42)

ER=oestrogen receptors; nodal ratio=number of involved nodes/number of dissected nodes; PR=progesterone receptors.

a ER/PR=‘1’ if positive and =‘0’ if negative.

b Predictive score=(nodal ratio × 3.05)+(tumour size × 0.15)−ER/PR × 1.15.

**Table 2:** Multivariate models

**Relapse-free survival**						**Overall survival**					
**Model 1**			**Model 2**			**Model 1**			**Model 2**		
**Variable**	**Odds ratio (95% CI)**	***P*-values**	**Variable**	**Odds ratio (95% CI)**	***P*-values**	**Variable**	**Odds ratio (95% CI)**	***P***-**values**	**Variable**	**Odds ratio (95% CI)**	***P*-values**
MVD^a^	2.7 (2–3.4)	0.005	MVD^a^	2.4 (1.7–3.1)	0.01	MVD^a^	3.0 (2.3–3.7)	0.002	MVD^a^	2.6 (1.9–3.3)	0.008
HER2^b^	2 (1.5–2.6)	0.001	HER2^b^	1.7 (1.2–2.3)	<0.05	HER2^b^	2.0 (1.4–2.5)	0.02	HER2^b^	1.66 (1.2–2.25)	<0.05
Score^c^	2.1 (1.6–2.7)	<0.05	Nodal ratio^d^	2.2 (1.7–2.8)	<0.01	Score^c^	2.2 (1.7–2.8)	0.005	Nodal ratio^d^	2.2 (1.6–2.8)	0.01
			Tumour size^e^	1.4 (0.95–2)	0.08				Tumour size^e^	1.5 (0.9–2.15)	0.1
			ER/PR^f^	0.29 (0.1–0.9)	<0.05				ER/PR^f^	0.3 (0.04–0.9)	<0.05

CI=confidence interval; ER=oestrogen receptors; MVD=microvessel density; PR=progesterone receptors.

a MVD: >14 *vs* ⩽14.

b HER2: positive *vs* negative.

c Predictive score (high *vs* low)=(nodal ratio × 3.05)+(tumour size × 0.15)−(ER/PR × 1.15). ER/PR=‘1’ if positive and=‘0’ if negative. Scores <2.41 are low, scores ⩾2.41 are high.

d Nodal ratio (⩾0.8 *vs* <0.8)=number of involved nodes/number of dissected nodes.

e Tumour size: ⩾5 *vs* <5 cm.

f ER/PR status: positive *vs* negative.
